# Use of [C_4_mim]Cl for efficient extraction of caffeoylquinic acids from sweet potato leaves

**DOI:** 10.1038/s41598-017-07291-9

**Published:** 2017-07-31

**Authors:** Toyonobu Usuki, Shingo Onda, Masahiro Yoshizawa-Fujita, Masahiro Rikukawa

**Affiliations:** Department of Materials and Life Sciences, Faculty of Science and Technology, Sophia University, 7-1 Kioicho, Chiyoda-ku, Tokyo 102-8554 Japan

## Abstract

Sweet potato, *Ipomoea batatas*, is a widely cultivated vegetable worldwide. The leaves contain polyphenolic natural products called caffeoylquinic acids (CQAs), which possess biological activities including inhibition of aggregation of amyloid peptides. The present study describes an efficient extraction and isolation procedure for CQAs from sweet potato leaves using a cellulose-dissolving ionic liquid. The results showed that, compared to methanol, use of 1-butyl-3-methylimidazolium chloride ([C_4_mim]Cl) allowed the extraction of a 6.5-fold greater amount of CQAs. This protocol will enable the efficient extraction of other organic compounds and biopolymers from natural materials.

## Introduction

**Figure 1:**
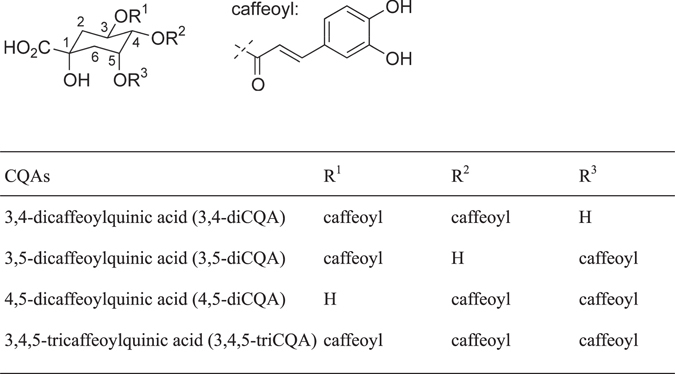
Structures of caffeoylquinic acids (CQAs).

Sweet potatoes, *Ipomoea batatas*, are cultivated as a vegetable in more than 100 countries, including China and African countries^1^. In general, after cultivation and harvest, portions of the roots are collected for food, while the leaves and stalks are discarded. However, the leaves are a good source of proteins, metals (including calcium, zinc, and iron), and natural products^1, 2^. In 2002, caffeoylquinic acids (CQAs, Fig. 1) were isolated from sweet potato leaves, and their structures were determined to be 3,4-dicaffeoylquinic acid (3,4-diCQA), 3,5-dicaffeoylquinic acid (3,5-diCQA), 4,5-dicaffeoylquinic acid (4,5-diCQA), and 3,4,5-tricaffeoylquinic acid (3,4,5-triCQA)^3^. CQAs have been shown to have anti-oxidant effects^4^, exhibit cytotoxicity^5^, and so on. Recently, it was also found that CQAs exert the potent inhibitions of the aggregation of amyloid beta peptides, which is known as one of the causes of Alzheimer’s disease^6^. Thus, sweet potato leaves are important resources for pharmacological, biomedical, and food chemical applications.

**Figure 2:**
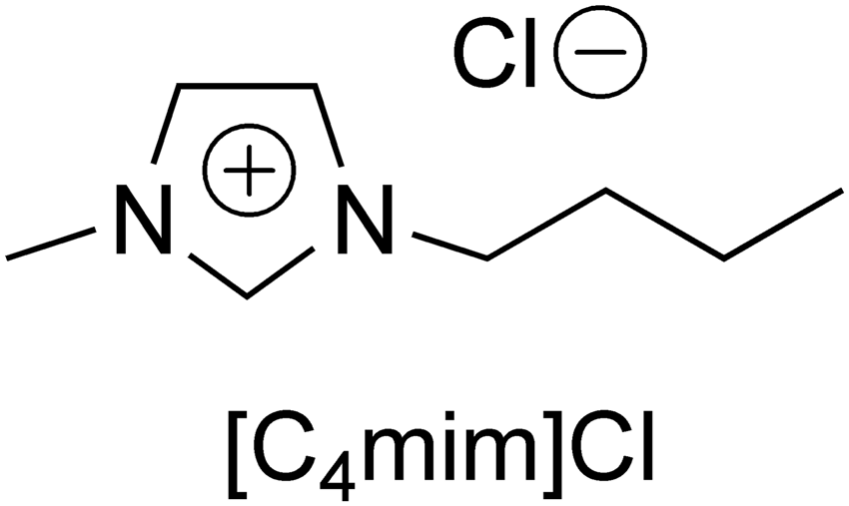
Structure of 1-butyl-3-methylimidazolium chloride ([C_4_mim]Cl).

Ionic liquids are salts with melting points of below 100 °C and have unique physicochemical properties, such as high thermal stability, negligible vapor pressure, and excellent capacity for solubilizing organic compounds and cellulose^7^. Therefore, ionic liquids have potential for the efficient extraction of natural products from plant leaves^8^. Among the ionic liquids that dissolve cellulose, 1-butyl-3-methylimidazolium chloride ([C_4_mim]Cl, Fig. 2) has an excellent cellulose-dissolving capacity that is greater at high temperature of above 100 °C^9^.

Recently, the efficient extraction and isolation of shikimic acid, the starting material in the commercial synthesis of oseltamivir phosphate, and bilobalide, a unique terpene trilactone isolated from *Ginkgo biloba* leaves, were reported using cellulose-dissolving ionic liquids^10, 11^. Meanwhile, extraction of essential oils from lemon grass and lemon myrtle using ionic liquids was also succeeded^12, 13^. Since a plant cell wall consists mainly of cellulose and hemicellulose, cellulose-dissoluble ionic liquids are able to extract plant natural products efficiently^14^ because most natural products in leaves are stored in vacuoles mainly as secondary metabolites. Thus, the goal of the present study was to develop a new method for the efficient extraction and isolation of CQAs from sweet potato leaves using a cellulose-dissoluble ionic liquid [C_4_mim]Cl^15^.

## Results and Discussion

First, extraction of CQAs from sweet potato leaves was conducted using methanol (MeOH) and water (H_2_O) for reference. The leaves used in this study were “Sui-Oh”, which is commercially available leaves used to make tea from the farm Sui-En (Fukuoka, Japan)^16^. The leaves of sweet potato were added to MeOH or H_2_O, respectively, and the solution was stirred under reflux for 1 hour. The resulting solution was then filtered to remove the residue of leaves. The filtrate was evaporated, and then the resulting extract was dissolved in H_2_O/MeOH (7:3 v/v). The solution was washed with *n*-hexane three times to remove undesired hydrophobic compounds. Removal of the organic solvent afforded crude extracts as a brownish solid.

Next, the extraction procedure using the cellulose-dissolving ionic liquid [C_4_mim]Cl was carried out as follows. The crushed sweet potato leaves were added to extraction solvents, and the solution was stirred for 1 hour: [C_4_mim]Cl at 150 °C, [C_4_mim]Cl/MeOH (1:1 w/w, 3:1 w/w) at 100 °C, [C_4_mim]Cl/H_2_O (1:1 w/w, 3:1 w/w) at 120 °C, and [C_4_mim]Cl/MeOH/H_2_O (2:1:1 w/w/w) at 100 °C. Then, MeOH was added to the solution, and the mixture was stirred at room temperature for 30 seconds. The solution was filtered, evaporated, and the extract was dissolved in H_2_O/MeOH (7:3 v/v). The solution was washed with *n*-hexane three times. Removal of organic solvents afforded the crude extract as a [C_4_mim]Cl solution.

**Figure 3:**
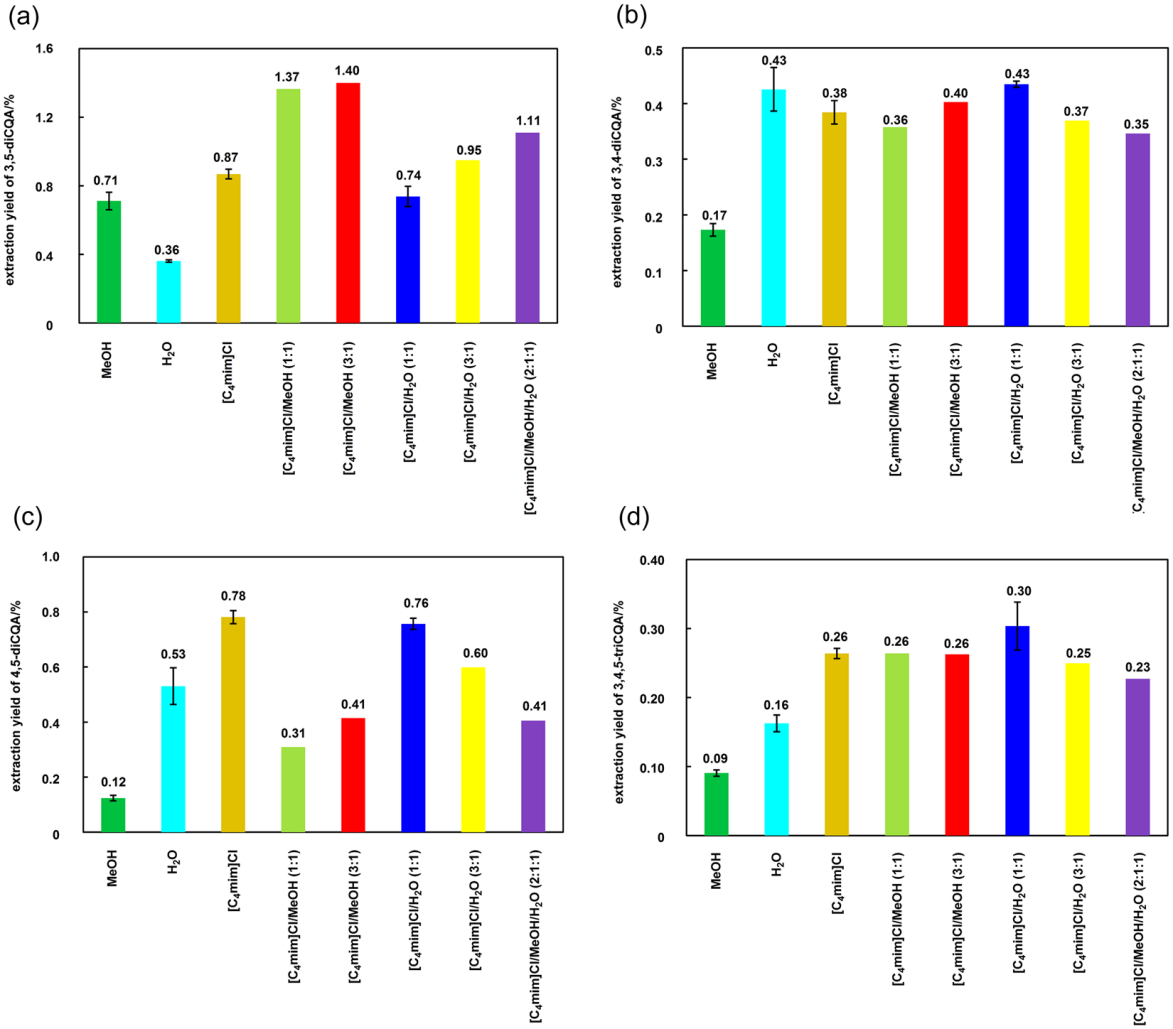
Extraction yields of (**a**) 3,4-diCQA, (**b**) 3,5-diCQA, (**c**) 4,5-diCQA, and (**d**) 3,4,5-triCQA. Experiments for MeOH (reflux), H_2_O (reflux), [C_4_mim]Cl (150 °C), and [C_4_mim]Cl/H_2_O (1:1 w/w, 120 °C) were performed three times and the average values are reported. Experiments for [C_4_mim]Cl/MeOH (1:1 w/w, 3:1 w/w, 100 °C), [C_4_mim]Cl/H_2_O (3:1 w/w, 120 °C), and [C_4_mim]Cl/MeOH/H_2_O (2:1:1 w/w/w, 100 °C) were carried out one time.

Quantitative analysis of the obtained extracts was performed using reversed-phase high performance liquid chromatography (RP-HPLC). For the analysis, the quantity of CQAs in individual extracts was calculated using a calibration curve. To prepare the calibration curves, 3,4-, 3,5-, and 4,5-diCQAs were purchased from a commercial supplier, whereas 3,4,5-triCQA was prepared by organic synthesis according to literature procedure starting from quinic acid^17^. Extraction yields were calculated as [amount of CQAs] per [original quantity of leaves], and the values for each CQA extracted using MeOH, [C_4_mim]Cl, [C_4_mim]Cl/MeOH, [C_4_mim]Cl/H_2_O, and [C_4_mim]Cl/MeOH/H_2_O are summarized in Fig. 3.

The extraction yields for 3,4-diCQA using H_2_O and [C_4_mim]Cl/H_2_O (1:1 w/w) were the same (0.43%), while the yield (0.40%) with [C_4_mim]Cl/MeOH (3:1 w/w) was 2.4 times greater than that obtained using MeOH (0.17%). The extraction yields for 3,5-diCQA using [C_4_mim]Cl/MeOH (3:1 w/w) and [C_4_mim]Cl/H_2_O (3:1 w/w) were 1.40% and 0.95%, respectively, which are 2.0 and 2.6 times greater than the yields achieved using MeOH (0.71%) and H_2_O (0.36%). The extraction yields for 4,5-diCQA using [C_4_mim]Cl/MeOH (3:1 w/w) and [C_4_mim]Cl/H_2_O (1:1 w/w) were 0.41% and 0.76%, respectively, which are 3.4 and 1.4 times higher than the yields achieved using MeOH (0.12%) and H_2_O (0.53%). Extraction of 4,5-diCQA with [C_4_mim]Cl afforded the best yield (0.78%), while yields for 3,4,5-triCQA using [C_4_mim]Cl/MeOH (1:1 w/w or 3:1 w/w) and [C_4_mim]Cl/H_2_O (1:1 w/w) were 0.26% and 0.30%, respectively, 2.9 and 1.9 times greater than those achieved using MeOH (0.09%) and H_2_O (0.16%). As the best comparison among the obtained results, the extraction yield of 4,5-diCQA using [C_4_mim]Cl (0.78%) was 6.5 times better than that using MeOH (0.12%).

Factors affecting the extraction results include the solubility of the cellulose making up the plant cell wall, the solubility of associated compounds in the extraction solvent, and the efficiency of stirring process. Because MeOH and H_2_O are poor solvents for cellulose, cellulose solubility decreases in a mixed solvent such as [C_4_mim]Cl/MeOH or [C_4_mim]Cl/H_2_O. However, this tendency did not affect overall extraction yields. The extraction yield for 3,5-diCQA using MeOH was much better than that using H_2_O, while the yields for other CQAs using H_2_O were much better than those using MeOH. This difference was probably due to the formation of micelle-like aggregation by CQAs except for 3,5-diCQA in H_2_O derived from the orientation of caffeoyl groups. The relatively long distance between the two caffeoyl groups in 3,5-diCQA may prevent the aggregation.

**Figure 4:**
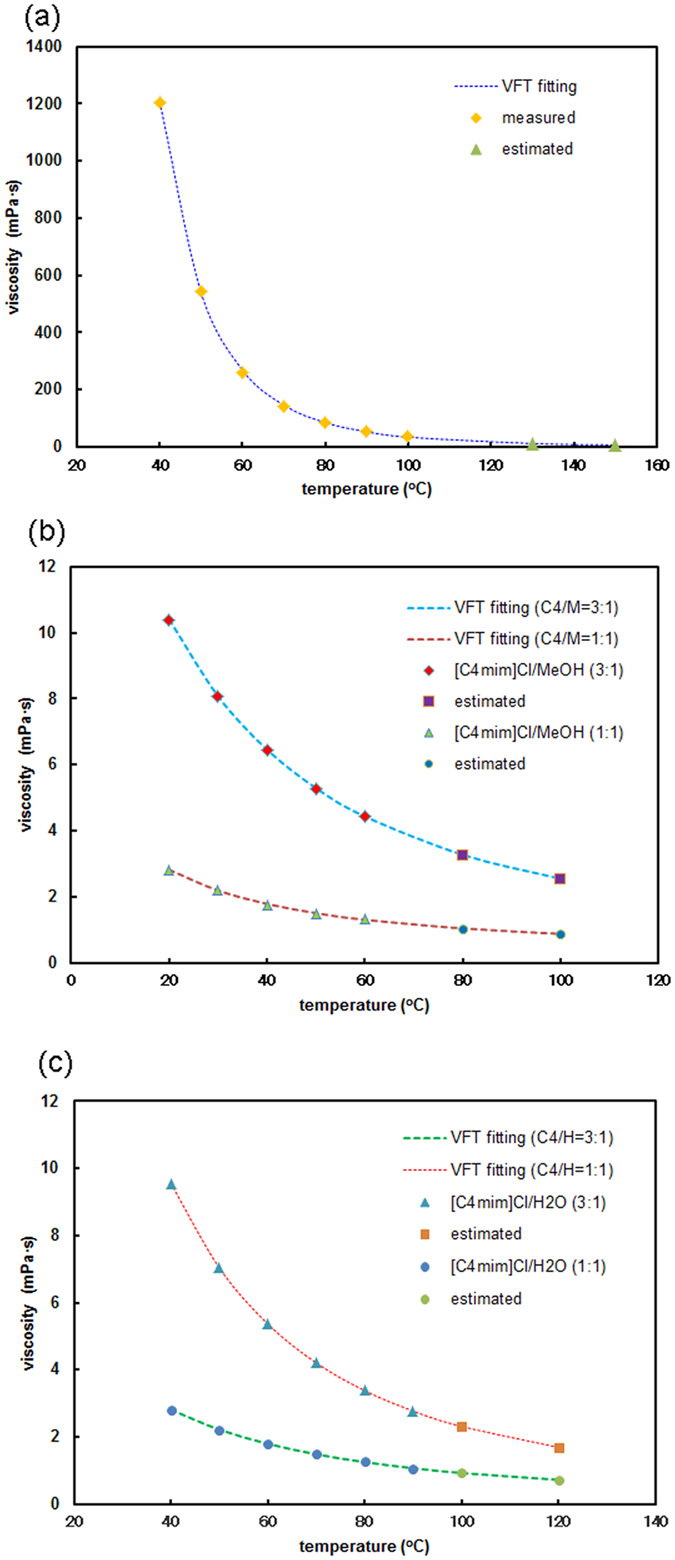
Viscosity of (**a**) [C_4_mim]Cl only (values at 130 and 150 °C estimated from VTF fitting^18^), (**b**) [C_4_mim]Cl/MeOH (1:1 w/w, 3:1 w/w, and values at 80 and 100 °C estimated from VTF fitting^18^), and (**c**) [C_4_mim]/H_2_O (1:1 w/w, 3:1 w/w, and values at 100 and 120 °C estimated from VTF fitting^18^).

To investigate the stirring efficiency, the viscosity of the extraction solvents was measured (Fig. 4). The ionic liquid [C_4_mim]Cl possessed extremely high viscosity as expected (Fig. 4a). A comparison between [C_4_mim]Cl/MeOH (1:1 w/w) and [C_4_mim]Cl/MeOH (3:1 w/w) indicated that greater amounts of [C_4_mim]Cl promoted greater viscosity (Fig. 4b), and a comparison between [C_4_mim]/H_2_O (1:1 w/w) and [C_4_mim]/H_2_O (3:1 w/w) also indicated the same tendency (Fig. 4c). Thus, the viscosity of a mixed solvent of [C_4_mim]Cl/MeOH or [C_4_mim]Cl/H_2_O dramatically decreased compared to that of [C_4_mim]Cl, which indicates that the stirring efficiency promoted an increase in the extraction yield using [C_4_mim]Cl/MeOH or [C_4_mim]Cl/H_2_O.

Furthermore, isolation of CQAs from the [C_4_mim]Cl layer was investigated using a salting-out and decantation technique. The [C_4_mim]Cl extract was added to a saturated sodium chloride solution, followed by addition of an extractipm solvent such as EtOAc/MeOH (10:1 v/v), EtOAc/MeOH (5:1 v/v), EtOAc/MeOH/HCO_2_H (100:10:1 v/v/v), or EtOAc/MeOH/HCO_2_H (100:20:1 v/v/v). The organic layer was collected from the mixture by decantation. This procedure was repeated four times, and the combined organic layers were removed to obtain the raw CQAs. The CQAs were subjected to HPLC analysis, and the amounts of CQAs obtained were compared to the original amounts of CQAs in [C_4_mim]Cl layer (Table 1).

**Table 1:** Extraction yield of CQAs from the [C_4_mim]Cl layer.

Entry	Extraction solvent	Extraction yield (%)			
		3,4-diCQA	3,5-diCQA	4,5-diCQA	3,4,5-triCQA
1	EtOAc/MeOH (10:1 v/v)	21	28	12	46
2	EtOAc/MeOH (5:1 v/v)	46	46	30	51
3	EtOAc/MeOH/HCO_2_H (100:10:1 v/v/v)	92	85	87	94
4	EtOAc/MeOH/HCO_2_H (100:20:1 v/v/v)	90	85	100	100

Extraction of CQAs from the [C_4_mim]Cl layer was attempted using an EtOAc/MeOH (10:1 v/v) extraction solvent (Table 1, entry 1), assuming a salting-out effect by saturated sodium chloride. However, the method afforded low yields with slight contamination of the [C_4_mim]Cl. An increase in MeOH promoted the solubility of the target compounds, resulting in an increase in the extraction yield (entry 2). To prevent the electrolytic dissociation of CQAs and increase the extraction efficiency, EtOAc/MeOH/HCO_2_H (100:10:1 v/v/v) was used as the extraction solvent. The results showed that most of the CQAs could be extracted from the [C_4_mim]Cl layer in 85 to 94% yields (entry 3). A change in the EtOAc/MeOH/HCO_2_H ratio to 100:20:1 v/v/v produced much better CQA extraction yields (entry 4). Because small amount of [C_4_mim]Cl was found in the resultant CQAs under the condition of entry 4, the condition of entry 3 would be the best for the extraction of CQAs from [C_4_mim]Cl layer. Thus, dissociation of a proton from formic acid probably prevented dissociation of protons from the CQAs, increasing the solubility in organic solvents (EtOAc).

## Conclusions

In summary, a method for the efficient extraction and isolation of CQAs from sweet potato leaves was developed using the cellulose-dissolving ionic liquid [C_4_mim]Cl. The use of [C_4_mim]Cl allowed the CQAs to be extracted more efficiently than using conventional MeOH or H_2_O solvents. The results demonstrated that the extraction yield of 4,5-diCQA using [C_4_mim]Cl (0.78%) was 6.5 times better than that using MeOH (0.12%). This method is expected to be applicable to the extraction and isolation of other organic compounds and biopolymers from natural materials.

## Experimental

### General methods

All reagents were obtained from commercial suppliers and used without further purification unless otherwise stated. The [C_4_mim]Cl and HPLC-grade acetonitrile were purchased from Sigma Aldrich (St. Louis, USA). Analytical thin layer chromatography (TLC) was performed on silica gel 60 F_254_ plates produced by Merck KGaA (Darmstadt, Germany). The HPLC-grade MeOH and HPLC-grade distilled H_2_O were purchased from Kanto Chemicals, Co. Ltd (Tokyo, Japan). Column chromatography was performed with acidic silica gel 60 (spherical, 40–50 μm) or neutral silica gel 60 N (spherical, 40–50 μm) produced by Kanto Chemicals, Co. Ltd (Tokyo, Japan). The HPLC standards, 3,4-dicaffeoylquinic acid (3,4-diCQA), 3,5-dicaffeoylquinic acid (3,5-diCQA), and 4,5-dicaffeoylquinic acid (4,5-diCQA), were purchased from ChromaDex^®^ Inc. (Irvine, USA). Sweet potato leaves (Sui-oh) were purchased from Shijoukai, Midorien (Fukuoka, Japan)^16^. The HPLC analyses were performed using a JASCO instrument equipped with a multiwavelength detector (MD-2010), semi-micro HPLC pump (PU-2085), autosampler (AS-2057), and column thermostat (CO-2060). Optical rotation was measured using a JASCO P-2200 digital polarimeter at the sodium D line (*λ* = 589 nm) and is reported as follows: [α]_D_^T^ (*c* g/100 mL, solvent). ^1^H nuclear magnetic resonance (NMR) spectra were recorded on a JEOL JNM-EXC 300 spectrometer. ^1^H NMR data are reported as follows: chemical shift (*δ*, ppm), integration, multiplicity (s, singlet; d, doublet; t, triplet; q, quartet; m, multiplet), coupling constants (*J*) in Hz, assignments. Densities were measured using a DMA38 instrument (Anton Paar K. G.) from 15 °C to 40 °C. Viscosities were measured using an automated micro viscometer (Anton Paar Gmbh) from 40 °C to 100 °C.

### Extraction of CQAs from sweet potato leaves

The CQAs were extracted from sweet potato leaves using MeOH or H_2_O as a reference. The sweet potato leaves (1 g) were treated with liquid nitrogen and then crushed into approximately 0.5 × 0.5 cm^2^ pieces in a mortar. Then, the crushed leaves were added to MeOH (16 g) or H_2_O (16 g), and the solution was stirred under reflux at around 80 °C or 120 °C, respectively, for 1 hour. The resulting solution was filtered with Celite 545 to remove the leaf residue. The filtrate was evaporated, and the extract was dissolved in H_2_O/MeOH (7:3 v/v). The solution was washed with *n*-hexane three times to remove undesired hydrophobic materials. Removal of the organic solvent afforded a crude extract as a brownish solid. Analyses of the extracts obtained were performed using RP-HPLC.

Extraction using the cellulose-dissolvable ionic liquid [C_4_mim]Cl was carried out as follows. Crushed sweet potato leaves (1 g) were added to the extraction solvents (16 g), such as [C_4_mim]Cl, [C_4_mim]Cl/MeOH (1:1 w/w, 3:1 w/w), [C_4_mim]Cl/H_2_O (1:1 w/w, 3:1 w/w), or [C_4_mim]Cl/MeOH/H_2_O (2:1:1 w/w/w), and the solution stirred under reflux for 1 hour at specific temperature: [C_4_mim]Cl at 150 °C, [C_4_mim]Cl/MeOH (1:1 w/w, 3:1 w/w) at 100 °C, [C_4_mim]Cl/H_2_O (1:1 w/w, 3:1 w/w) at 120 °C, and [C_4_mim]Cl/MeOH/H_2_O (2:1:1 w/w/w) at 100 °C. Then, MeOH (50–80 mL) was added to the solution, and the mixture was stirred at room temperature for 30 seconds. The solution was filtered with Celite 545, the filtrate was evaporated using a rotary evaporator, and then the extract was dissolved in H_2_O/MeOH (7:3 v/v). The solution was washed with *n*-hexane three times. After evaporation, the crude extract derived from an ionic liquid method was obtained as a [C_4_mim]Cl solution. Analyses of the extracts obtained were performed on RP-HPLC.

### HPLC analysis of CQAs

Quantitative analyses of the extracts obtained were performed using an RP-HPLC system [column: YMC-Pack ODS-AM (150 × 4.6 mm); mobile phase: 0.2% formic acid aqueous solution/acetonitrile (gradient), flow rate: 1.0 mL/min; detection: 326 nm; injection amount: 10 μL; temperature: 40 °C]^3^. The quantity of CQA from individual extraction experiments was calculated using a calibration curve. The equations 1–4 for each CQA were obtained using the formulas below, where *x* represents peak area and *y* represents the amount of CQA (mg):

1 $$3,4 \mbox{-} \mathrm{diCQA}:y=2.860\times {10}^{-10}x+1.360\times {10}^{-4}\,({{\rm{R}}}^{2}=0.9968)$$

2 $$3,5 \mbox{-} \mathrm{diCQA}:y=3.079\times {10}^{-10}x-8.534\times {10}^{-5}\,({{\rm{R}}}^{2}=0.9991)$$

3 $$4,5 \mbox{-} \mathrm{diCQA}:y=3.941\times {10}^{-10}x+1.164\times {10}^{-4}\,({{\rm{R}}}^{2}=0.9966)$$

4 $$3,4,5 \mbox{-} \mathrm{triCQA}:y=6.549\times {10}^{-10}x+2.420\times {10}^{-5}\,({{\rm{R}}}^{2}=0.9998)$$

The extraction yields were calculated as the [quantity of CQAs] per [original quantity of leaves].

### Synthesis of 3,4,5-triCQA

The 3,4,5-triCQA was synthesized starting from quinic acid^17^. 3,4,5-Triacety-lcaffeoyl-1,7-isopropyridenequinide was synthesized using a previously reported method^17^. To a solution of 3,4,5-triacetylcaffeoyl-1,7-isopropyridenequinide (12 mg, 0.012 mmol, 1.0 eq) in THF (1 mL) was added 2 M HCl (1 mL, 1.1 mmol, 94 eq). After stirring at room temperature for 11 days, the reaction mixture was concentrated *in vacuo*. Purification by HPLC afforded **1** as a colorless solid (2.2 mg, 0.0032 mmol, 26%); [α]_D_^25^ −343 (*c* 0.13, MeOH) [ref. 15: [α]_D_^25^ −314 (*c* 1.0, MeOH)]; ^1^H NMR (300 MHz, CD_3_OD) *δ*7.61 (1H, d, *J* = 15.6 Hz), 7.54 (1H, d, *J* = 15.9 Hz), 7.51 (1H, d, *J* = 15.9 Hz), 7.08–7.03 (1H, m), 7.02–6.97 (2H, m), 6.94–6.90 (2H, m), 6.85–6.79 (1H, m), 6.78–6.68 (2H, m), 6.35 (1H, d, *J* = 15.9 Hz), 6.21 (1H, d, *J* = 15.9 Hz), 6.19 (1H, d, *J* = 15.6 Hz), 5.68 (2H, m), 5.31 (1H, dd, *J* = 8.7, 3.0 Hz), 2.34–2.11 (4H, m).

### Extraction of CQAs from [C_4_mim]Cl layer

A total of 10 g of [C_4_mim]Cl extract was added to 100 mL of saturated sodium chloride solution, and the solution was stirred for less than 1 min. The extraction solvent (150 mL), EtOAc/MeOH (10:1 v/v), EtOAc/MeOH (5:1 v/v), EtOAc/MeOH/HCO_2_H (100:10:1 v/v/v), or EtOAc/MeOH/HCO_2_H (100:20:1 v/v/v), then was added to the mixture, and the resulting mixture was stirred for 5 min. The organic layer was collected from the mixture by decantation. This procedure was repeated four times, and the combined organic layers were evaporated to obtain the crude CQA. The resultant CQAs were subjected to HPLC analysis, and the amount of CQA obtained was compared to the original amounts in the [C_4_mim]Cl layer.

### Densimetry of [C_4_mim]Cl and its co-solvents

Densities of [C_4_mim]Cl, [C_4_mim]Cl/MeOH (1:1, 3:1, w/w), [C_4_mim]Cl/H_2_O (1:1, 3:1, w/w), and [C_4_mim]Cl/MeOH/H_2_O (2:1:1 w/w/w) were measured using a DMA 38 instrument (Anton Paar K. G.) at temperatures from 15 °C to 40 °C. Theoretical densities at temperatures above 50 °C were calculated using individual calibration curves.

### Viscometry of [C_4_mim]Cl and its co-solvents

Viscosities of [C_4_mim]Cl, [C_4_mim]Cl/MeOH (1:1, 3:1, w/w), and [C_4_mim]Cl/H_2_O (1:1, 3:1, w/w) were measured using an automated micro viscometer (Anton Paar K. G.). Theoretical viscosities for rest temperature were calculated using the VFT equation^18^.

## Electronic supplementary material

Supplementary Information
