# Supplementary material for: Use of [C4mim]Cl for efficient extraction of caffeoylquinic acids from sweet potato leaves
Source: Sci Rep. 2017 Jul 31;7:6890. doi: 10.1038/s41598-017-07291-9 (PMC5537367; doi:10.1038/s41598-017-07291-9)
Supplement: Supplementary file 1 — Supplementary Information [file 41598_2017_7291_MOESM1_ESM.pdf]

Use of [C_4mim]Cl for efficient extraction of caffeoylquinic acids from sweet potato leaves

Toyonobu Usuki,* Shingo Onda, Masahiro Yoshizawa-Fujita, and Masahiro Rikukawa

Department of Materials and Life Sciences, Faculty of Science and Technology, Sophia University, 7-1 Kioicho, Chiyoda-ku, Tokyo 102-8554, Japan

*Corresponding author. T. Usuki

E-mail: t-usuki@sophia.ac.jp, Tel.: +81 3 3238 3446

1. Calibration curve by HPLC analysis of natural 3,4-diCQA:

In order to prepare the calibration curve for 3,4-diCQA, HPLC analysis of natural 3,4-diCQA (0.022 mg/mL, 0.074 mg/mL, 0.22 mg/mL, and 0.67 mg/mL) was performed as shown in **Figure S1** as the example under the condition described as main text. HPLC charts were acquired on a JASCO's instrument. Since the obtained peak area of 3,4-diCQA was 665773, 1880869, 6441890, and 23081381, respectively, amount of 3,4-diCQA could be plotted as shown in **Figure S2**. Consequently, the fitting for the plots gave an equation described as $y = 2.860 \times 10^{-10} x + 1.360 \times 10^{-4}$ (y: amout of 3,4-diCQA (mg), x: peak area) with $R^2 = 0.9968$ for the quantitative analysis of 3,4-diCQA.

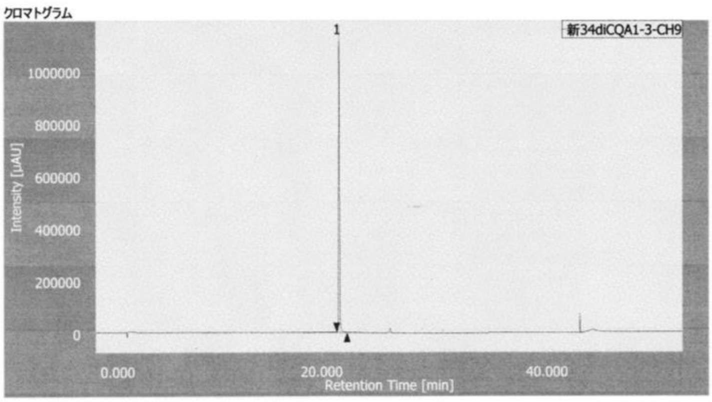

クロマトグラム情報
ユーザー名 usuki
更新日時 2014/03/12 17:23:33
コメント
HPLC システム名 HPLC(AS)
測定日 2014/03/12 15:33:45
注入量 10.00 [μL]
サンプル# 27
プロジェクト名 HPLC-1
取込時間 52.0 [min]
測定シーケンス 0312 新 34-diCQA 1-3 1-9
コントロールメソッド CQA アセニト ギ酸 YMC 4.6id 52min 1.0ml
ピーク ID テーブル
検量線テーブル
追加情報

ピーク情報

#	ピーク名	CH	NTP	分離度	シンメトリー係数	警告	補正定量値	ISTD ピーク名	ISTD 添加量	定量ファクター	ピークスタート	ピークエンド
1	Unknown	9	320903	N/A	1.191		N/A	N/A	N/A	1.0000	21.333	22.253

#	BL 開始	BL 終了	ピーク幅	S/N	分離係数	キャパシティー比	検出方法	計算値	ピークファクター	真値	重量	判定	面積 [μV·sec]
1	18.373	22.733	0.090	N/A	N/A	N/A	オートマチック	N/A	N/A	N/A	0.00000	N/A	6441890

#	tR [min]	高さ [μV]	面積%	高さ%	定量値
1	21.547	1132772	100.000	100.000	N/A

Figure S1. HPLC chart of 3,4-diCQA (0.22 mg/mL). The condition is described in main text.

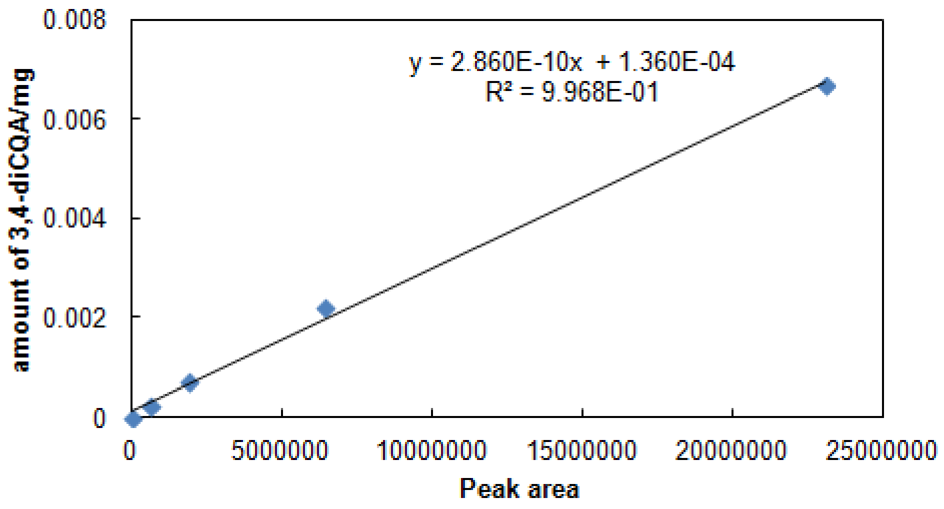

Figure S2. Calibration curve of 3,4-diCQA based on the HPLC analysis.

2. Calibration curve by HPLC analysis of natural 3,5-diCQA:

In order to prepare the calibration curve for 3,5-diCQA, HPLC analysis of natural 3,5-diCQA (0.022 mg/mL, 0.074 mg/mL, 0.22 mg/mL, and 0.67 mg/mL) was performed as shown in **Figure S3** as the example under the condition described as main text. HPLC charts were acquired on a JASCO's instrument. Since the obtained peak area of 3,5-diCQA was 854824, 2891934, 7861714, and 21775409, respectively, amount of 3,5-diCQA could be plotted as shown in **Figure S4**. Consequently, the fitting for the plots gave an equation described as $y = 3.079 \times 10^{-10} x - 8.534 \times 10^{-5}$ (y: amout of 3,5-diCQA (mg), x: peak area) with $R^2 = 0.9991$ for the quantitative analysis of 3,5-diCQA.

Figure S3. HPLC chart of 3,5-diCQA (0.22 mg/mL). The condition is described in main text.

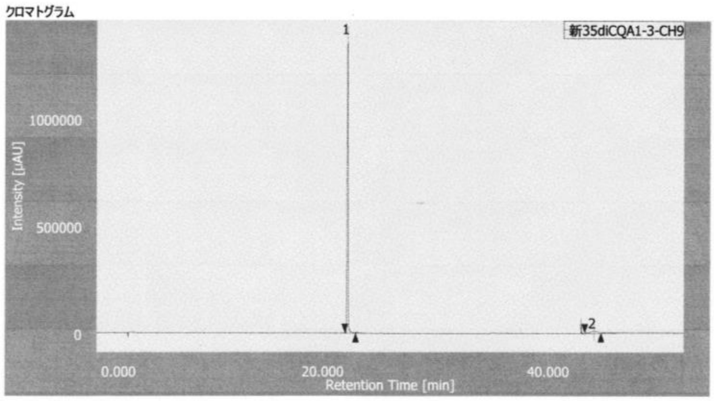

クロマトグラム情報

ユーザー名	usuki
更新日時	2014/03/13 17:54:29
コメント	
HPLC システム名	HPLC(AS)
測定日	2014/03/13 12:24:17
注入量	10.00 [µL]
サンプル#	17
プロジェクト名	HPLC-1
取込時間	52.0 [min]
測定シーケンス	CQA0313-新 35-diCQA 1-1 3 9
コントロールメソッド	CQA アセニト ギ酸 YMC 4.6id 52min 1.0ml
ピーク ID テーブル	
検量線テーブル	
追加情報	

ピーク情報

#	ピーク名	CH	NTP	分離度	シンメトリー係数	警告	補正定量値	ISTD ピーク名	ISTD 添加量	定量ファクター	ピークスタート	ピークエンド
1	Unknown	9	379975	23.582	1.230		N/A	N/A	N/A	1.0000	21.960	22.907
2	Unknown	9	10544	N/A	N/A		N/A	N/A	N/A	1.0000	43.213	44.640

#	BL 開始	BL 終了	ピーク幅	S/N	分離係数	キャパシティー比	検出方法	計算値	ピークファクター	真値	重量	判定	面積 [µV·sec]
1	21.173	24.053	0.085	N/A	N/A	N/A	オートマチック	N/A	N/A	N/A	0.00000	N/A	7861714
2	42.987	46.467	1.010	N/A	N/A	N/A	オートマチック	N/A	N/A	N/A	0.00000	N/A	660013

#	tR [min]	高さ [µV]	面積%	高さ%	定量値
1	22.173	1387820	92.255	99.138	N/A
2	44.040	12064	7.745	0.862	N/A

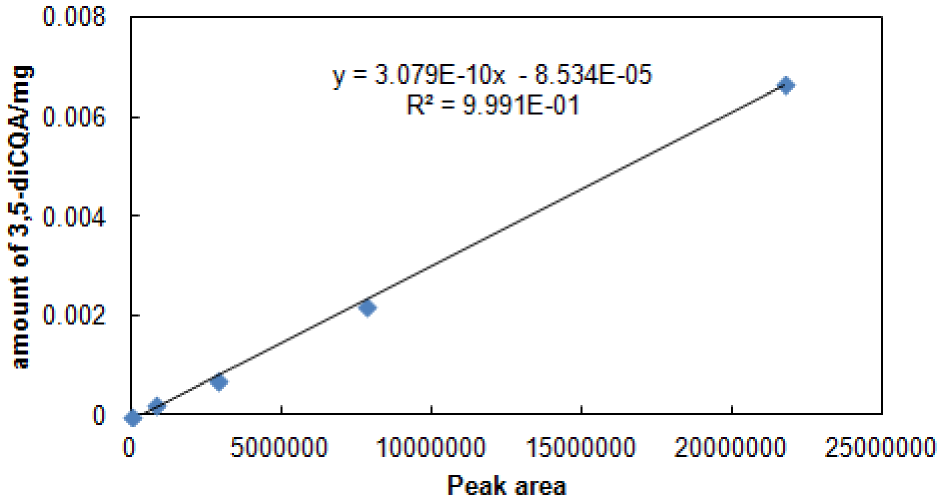

Figure S4. Calibration curve of 3,5-diCQA based on the HPLC analysis.

3. Calibration curve by HPLC analysis of natural 4,5-diCQA:

In order to prepare the calibration curve for 4,5-diCQA, HPLC analysis of natural 4,5-diCQA (0.022 mg/mL, 0.074 mg/mL, 0.22 mg/mL, and 0.67 mg/mL) was performed as shown in **Figure S5** as the example under the condition described as main text. HPLC charts were acquired on a JASCO's instrument. Since the obtained peak area of 4,5-diCQA was 465187, 1615250, 4633030, and 16807729, respectively, amount of 4,5-diCQA could be plotted as shown in **Figure S6**. Consequently, the fitting for the plots gave an equation described as $y = 3.941 \times 10^{-10} x + 1.164 \times 10^{-4}$ (y: amout of 4,5-diCQA (mg), x: peak area) with $R^2 = 0.9966$ for the quantitative analysis of 4,5-diCQA.

クロマトグラム

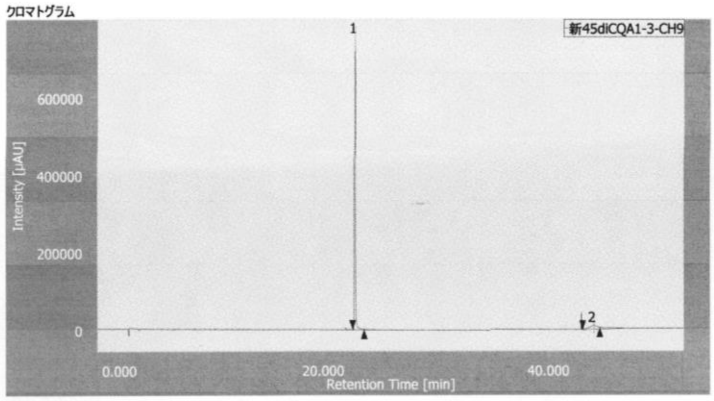

クロマトグラム情報

ユーザー名	usuki
更新日時	2014/03/13 17:54:32
コメント	
HPLC システム名	HPLC(AS)
測定日	2014/03/13 15:06:31
注入量	10.00 [μL]
サンプル#	22
プロジェクト名	HPLC-1
取込時間	52.0 [min]
測定シーケンス	CQA0313-新 45-diCQA 1-1 3 9
コントロールメソッド	CQA アセニト ギ酸 YMC 4.6id 52min 1.0ml
ピーク ID テーブル	
検量線テーブル	
追加情報	

ピーク情報

#	ピーク名	CH	NTP	分離度	シンメトリー係数	警告	補正定量値	ISTD ピーク名	ISTD 添加量	定量ファクター	ピークスタート	ピークエンド
1	Unknown	9	336592	25.027	1.202		N/A	N/A	N/A	1.0000	22.627	23.627
2	Unknown	9	13036	N/A	N/A		N/A	N/A	N/A	1.0000	43.013	44.520

#	BL 開始	BL 終了	ピーク幅	S/N	分離係数	キャパシティー比	検出方法	計算値	ピークファクター	真値	重量	判定	面積 [μV·sec]
1	21.467	24.840	0.092	N/A	N/A	N/A	オートマチック	N/A	N/A	N/A	0.00000	N/A	4633030
2	43.013	46.747	0.907	N/A	N/A	N/A	オートマチック	N/A	N/A	N/A	0.00000	N/A	574898

#	tR [min]	高さ [μV]	面積%	高さ%	定量値
1	22.800	762648	88.961	98.508	N/A
2	44.000	11548	11.039	1.492	N/A

Figure S5. HPLC chart of shikimic acid (0.22 mg/mL). The condition is described in main text.

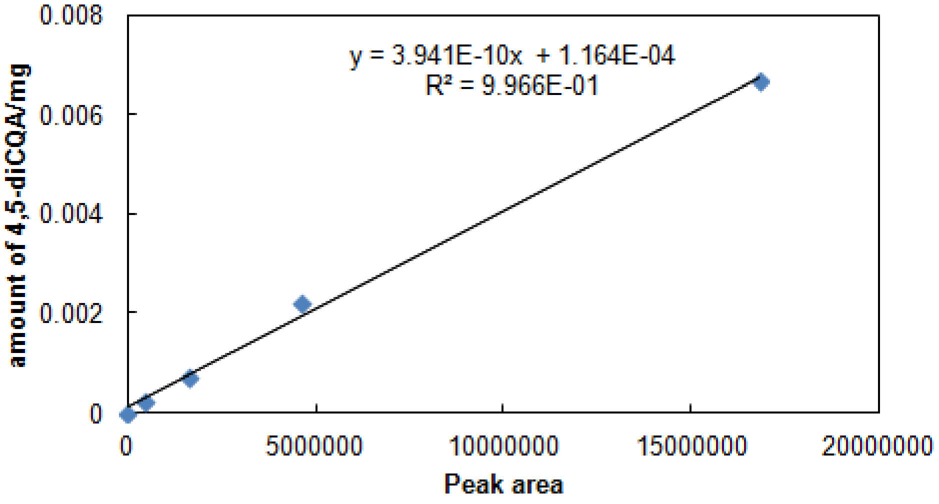

Figure S6. Calibration curve of shikimic acid based on the HPLC analysis.

4. Calibration curve by HPLC analysis of synthetic 3,4,5-triCQA:

In order to prepare the calibration curve for 3,4,5-triCQA, HPLC analysis of synthetic 3,4,5-triCQA (0.010 mg/mL, 0.030 mg/mL, 0.089 mg/mL, and 0.27 mg/mL) was performed as shown in **Figure S7** as the example under the condition described as main text. HPLC charts were acquired on a JASCO's instrument. Since the obtained peak area of 3,4,5-triCQA was 143499, 414083, 1228163, and 4061847, respectively, amount of 3,4,5-triCQA could be plotted as shown in **Figure S8**. Consequently, the fitting for the plots gave an equation described as $y = 6.549 \times 10^{-10} x + 2.420 \times 10^{-5}$ (y: amout of 3,4,5-triCQA (mg), x: peak area) with $R^2 = 0.9998$ for the quantitative analysis of 3,4,5-triCQA.

クロマトグラム

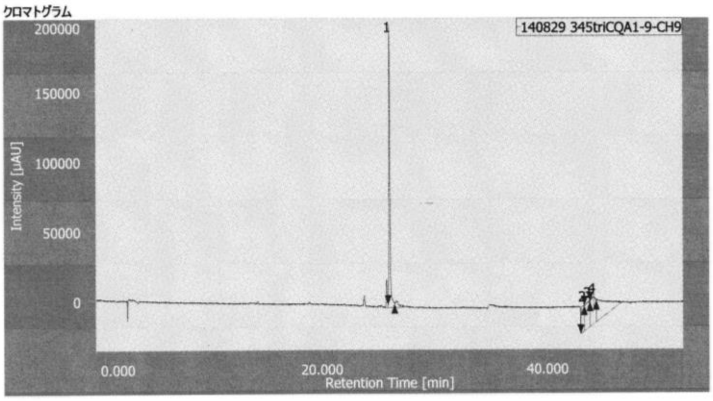

クロマトグラム情報

ユーザー名	usuki
更新日時	2014/08/29 11:26:17
コメント	
HPLC システム名	HPLC(AS)
測定日	2014/08/29 10:34:14
注入量	10.00 [µL]
サンプル#	13
プロジェクト名	HPLC-1
取込時間	52.0 [min]
測定シーケンス	20140829 241 345triCQA 検量線 1-9 52min
コントロールメソッド	CQA アセニト ギ酸 YMC 4.6id 52min 1.0ml
ピーク ID テーブル	
検量線テーブル	
追加情報	

ピーク情報

#	ピーク名	CH	NTP	分離度	シンメトリー係数	警告	補正定量値	ISTD ピーク名	ISTD 添加量	定量ファクター	ピークスタート	ピークエンド
1	Unknown	9	521036	N/A	1.563		N/A	N/A	N/A	1.0000	25.867	26.440
2	Unknown	9	N/A	N/A	N/A		N/A	N/A	N/A	1.0000	42.920	43.253
3	Unknown	9	N/A	N/A	N/A		N/A	N/A	N/A	1.0000	43.253	43.733
4	Unknown	9	N/A	N/A	N/A		N/A	N/A	N/A	1.0000	43.733	44.307

#	BL 開始	BL 終了	ピーク幅	S/N	分離係数	キャパシティー比	検出方法	計算値	ピークファクター	真値	重量	判定	面積 [µV·sec]
1	25.507	27.320	0.085	N/A	N/A	N/A	オートマチック	N/A	N/A	N/A	0.00000	N/A	1228163
2	42.920	46.493	N/A	N/A	N/A	N/A	オートマチック	N/A	N/A	N/A	0.00000	N/A	349917
3	42.920	46.493	N/A	N/A	N/A	N/A	オートマチック	N/A	N/A	N/A	0.00000	N/A	534455
4	42.920	46.493	N/A	N/A	N/A	N/A	オートマチック	N/A	N/A	N/A	0.00000	N/A	634101

#	tR [min]	高さ [µV]	面積%	高さ%	定量値
1	25.987	196445	44.715	77.303	N/A
2	43.227	19118	12.740	7.523	N/A
3	43.680	19141	19.459	7.532	N/A
4	44.067	19420	23.086	7.642	N/A

Figure S7. HPLC chart of 3,4,5-triCQA (0.089 mg/mL). The condition is described in main text.

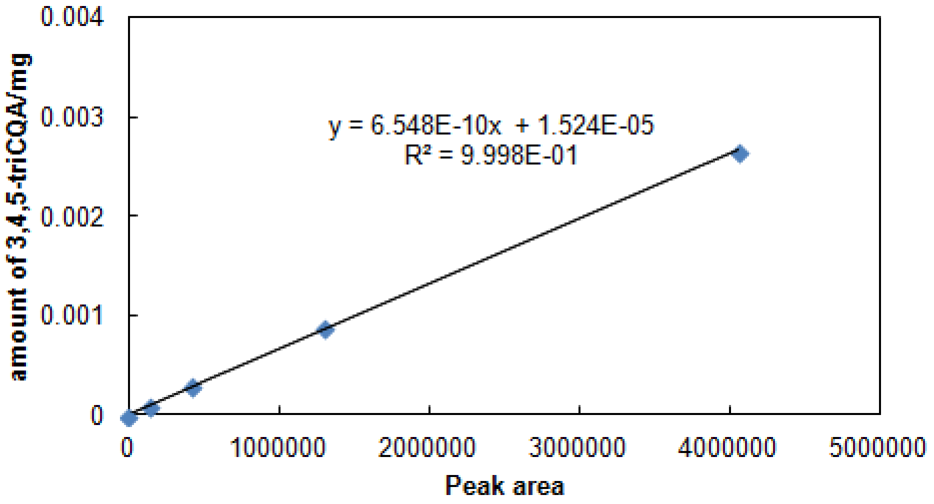

Figure S8. Calibration curve of 3,4,5-triCQA based on the HPLC analysis.

5. HPLC analysis of extracted 3,4-diCQA by MeOH:

When 0.137 mg of the extract (total: 280 mg) by methanol from 1.23 g of sweet potato leaves was injected into the HPLC, the chart was obtained as **Figure S9** under the condition described as main text. Since the peak area of 3,4-diCQA was found to be 718488, the value was substituted for *y* in the equation of $y = 2.860 \times 10^{-10} x + 1.360 \times 10^{-4}$, resulting in estimation of amount of 3,4-diCQA "*y*" as 9.77×10^{-2} mg. Because total amount of 3,4-diCQA in 280 mg of the extract was calculated to be 2.00 mg (= 9.77×10^{-2} mg × 280 mg / 0.137 mg), we found the extraction yield of 3,4-diCQA by MeOH was to be 0.162 % (= 2.00 mg / 1.23 g × 100). Extraction yields of 3,4-diCQA, 3,5-diCQA, 4,5-diCQA and 3,4,5-triCQA by MeOH, [C_4mim]Cl/MeOH (1:1, 3:1), [C_4mim]Cl/H_2O (1:1, 3:1) and [C_4mim]Cl/MeOH/H_2O (2:1:1) were calculated in the same procedures.

クロマトグラム

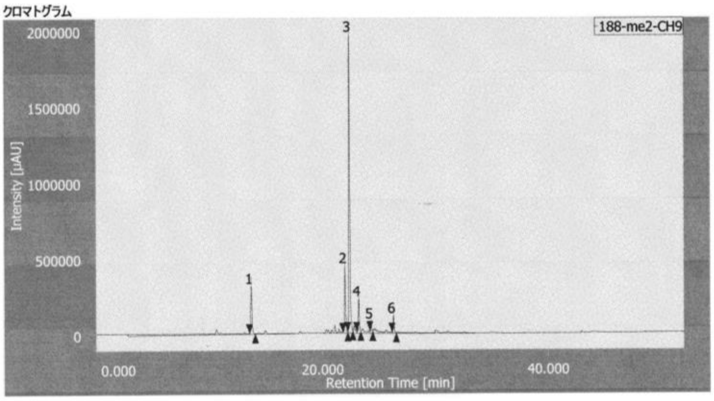

クロマトグラム情報

ユーザー名	usuki
更新日時	2013/10/07 19:15:23
コメント	
HPLC システム名	HPLC(AS)
測定日	2013/10/07 16:18:48
注入量	10.00 [µL]
サンプル#	21
プロジェクト名	HPLC−1
取込時間	52.0 [min]
測定シーケンス	CQA1007 YMC 188 me 2
コントロールメソッド	CQA アセニト ギ酸 YMC 4.6id 52min 1.0ml
ピーク ID テーブル	
検量線テーブル	
追加情報	

ピーク情報

#	ピーク名	CH	NTP	分離度	シンメトリー係数	警告	補正定量値	ISTD ピーク名	ISTD 添加量	定量ファクター	ピークスタート	ピークエンド
1	Unknown	9	75088	45.789	1.140		N/A	N/A	N/A	1.0000	13.520	14.040
2	Unknown	9	291711	2.524	1.396		N/A	N/A	N/A	1.0000	21.787	22.227
3	Unknown	9	292048	5.077	1.180		N/A	N/A	N/A	1.0000	22.227	22.667
4	Unknown	9	355994	6.551	N/A		N/A	N/A	N/A	1.0000	23.027	23.373
5	Unknown	9	294271	11.761	N/A		N/A	N/A	N/A	1.0000	24.187	24.440
6	Unknown	9	433774	N/A	1.188		N/A	N/A	N/A	1.0000	26.133	26.507

#	BL 開始	BL 終了	ピーク幅	S/N	分離係数	キャパシティー比	検出方法	計算値	ピークファクター	真値	重量	判定	面積 [µV·sec]
1	2.760	17.493	0.118	N/A	N/A	N/A	オートマチック	N/A	N/A	N/A	0.00000	N/A	2434270
2	19.947	33.573	0.096	N/A	N/A	N/A	オートマチック	N/A	N/A	N/A	0.00000	N/A	2938973
3	19.947	33.573	0.098	N/A	N/A	N/A	オートマチック	N/A	N/A	N/A	0.00000	N/A	13210949
4	19.947	33.573	0.092	N/A	N/A	N/A	オートマチック	N/A	N/A	N/A	0.00000	N/A	1447521
5	19.947	33.573	0.105	N/A	N/A	N/A	オートマチック	N/A	N/A	N/A	0.00000	N/A	576162
6	19.947	33.573	0.094	N/A	N/A	N/A	オートマチック	N/A	N/A	N/A	0.00000	N/A	755261

#	tR [min]	高さ [µV]	面積%	高さ%	定量値
1	13.693	312117	11.395	9.941	N/A
2	21.973	440586	13.757	14.032	N/A
3	22.387	1972281	61.840	62.815	N/A
4	23.200	223177	6.776	7.108	N/A
5	24.293	77739	2.697	2.476	N/A
6	26.280	113915	3.535	3.628	N/A

Figure S9. HPLC chart of extracted CQAs by MeOH. The condition is described in main text.
